# Effect of electroacupuncture on inhibition of inflammatory response and oxidative stress through activating ApoE and Nrf2 in a mouse model of spinal cord injury

**DOI:** 10.1002/brb3.2328

**Published:** 2021-08-22

**Authors:** Ni Dai, Chenglin Tang, Hui Liu, Siqin Huang

**Affiliations:** ^1^ Traditional Chinese Medicine College Chongqing Medical University Chongqing China; ^2^ Institute of Neuroscience Chongqing Medical University Chongqing China

**Keywords:** apolipoprotein E, electroacupuncture, inflammatory response, nuclear factor erythroid 2‐related factor 2, oxidative stress, spinal cord injury

## Abstract

**Introduction:**

Electroacupuncture protects neurons and myelinated axons after spinal cord injury by mitigating the inflammatory response and oxidative stress, but how it exerts these effects is unclear.

**Methods and results:**

Spinal cord injury was induced in C57BL/6 wild‐type and apolipoprotein E (ApoE) knockout (*ApoE* ^–/–^) mice, followed by electroacupuncture or ApoE mimetic peptide COG112 treatment. Mice with spinal cord injury suffered loss of myelinated axons and hindlimb motor function through the detections of Basso mouse scale, histology, and transmission electron microscopy; electroacupuncture partially reversed these effects in wild‐type mice but not in ApoE^–/–^ mice. Combining exogenous ApoE administration with electroacupuncture significantly mitigated the effects of spinal cord injury in both mouse strains, and these effects were associated with up‐regulation of anti‐inflammatory cytokines and down‐regulation of pro‐inflammatory cytokines which were detected by quantitative reverse transcription‐polymerase chain reaction. Combination treatment also reduced oxidative stress by up‐regulating ApoE and Nrf2/HO‐1 signaling pathway through the detections of immunofluorescence and western blot analysis.

**Conclusions:**

These results suggest that electroacupuncture protects neurons and myelinated axons following spinal cord injury through an ApoE‐dependent mechanism.

## Graphical abstract

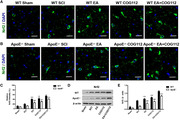

## 1 INTRODUCTION

Spinal cord injury (SCI) leads to irreversible sensory, motor, and autonomic dysfunction. It places a substantial financial and emotional burden on individuals and societies around the world (GBD 2016 Traumatic Brain Injury and Spinal Cord Injury Collaborators, 2019; Tator & Fehlings, 1991). After primary injury (irreversible mechanical injury), secondary processes including inflammatory response, oxidative stress, neuronal apoptosis, and axonal demyelination irreversibly damage the spinal cord (Oyinbo, 2011). Particularly important in SCI are inflammatory responses and oxidative stress (Silva et al., 2014).

To date, there is still no treatment for the injured spinal cord (Assinck et al., 2017; McDonald & Sadowsky, 2002; Tsata & Wehner, 2021). However, rapid medical treatments can reduce secondary damage, protect neurons that survive primary damage, and improve subsequent functional recovery at the early stage of SCI (Ahuja & Fehlings, 2016). Some adjunctive therapies are used as an effective treatment of acute SCI clinically, such as neuroprotectant agents (Garcia et al., 2016) and electroacupuncture (EA; Alvarado‐Sanchez et al., 2019).

EA is widely used in various acute and chronic diseases, and has shown therapeutic efficacy against central nervous system diseases, especially SCI (Liu et al., 2010; Yan et al., 2011). EA accelerates neural function recovery after SCI by reducing neuronal cell apoptosis (Jin et al., 2019), prompting neural stem cells or precursor cells to proliferate and differentiate into neurons (Ding et al., 2013), and accelerating axon regeneration and remyelination (Huang et al., 2015). EA may achieve these outcomes by shifting macrophages from a pro‐inflammatory M1 phenotype to an anti‐inflammatory M2 phenotype, leading to down‐regulation of pro‐inflammatory cytokines such as tumor necrosis factor (TNF)‐α, interleukin (IL)‐6, and IL‐1β (Zhao et al., 2017). Simultaneous up‐regulation of the anti‐oxidant enzymes superoxide dismutase and glutathione peroxidase by EA may also play a role in its efficacy (Yu et al., 2010). EA may even inhibit the nuclear factor kappa B (NF‐κB) signaling pathway (He et al., 2019), which is consistent with our previous work that EA can reduced inflammation through inhibiting IL‐1β and NF‐κB expression in C57BL/6 wild‐type mice (Dai et al., 2019). Whether these mechanisms allow EA to mitigate the inflammatory response and oxidative stress after SCI has not been well investigated.

Apolipoprotein E (ApoE) is a major apolipoprotein for the regulation of lipid and cholesterol metabolism in central nervous system (CNS; Mahley, 1988), and has been associated with physiopathology of Alzheimer's (Griffin et al., 2019) and atherosclerosis (Rasmiena et al., 2015). ApoE exerts anti‐inflammatory, anti‐oxidative and anti‐apoptotic effects in CNS (Jiang et al., 2015; Kitagawa et al., 2002; K. Li et al., 2015)^,^ as well as promotion of axonal regeneration and remyelination after a nerve injury (Vance et al., 2000). Deficiency of ApoE results in exaggerated inflammatory response, reduces anti‐oxidants levels, and prompts neuronal apoptosis after SCI in rats (Lomnitski et al., 1997; Yang et al., 2018). Treatment with exogenous ApoE can ameliorate motor deficit and tissue damage as well as modify the inflammatory response (R. Wang et al., 2014). As we found in the previous work, the up‐regulation of ApoE by EA can reduce inflammation and oxidative stress reactions (Dai et al., 2019). ApoE is therefore considered to play a neuroprotective function (Cheng et al., 2018).

ApoE may exert neuroprotective effects in part by interacting with nuclear factor erythroid 2‐related factor (Nrf2), which modulates the expression of genes involved in the cellular anti‐inflammatory and anti‐oxidant responses (Loboda et al., 2016; Samarghandian et al., 2020). Nrf2 is activated by oxidative stress after injury (Baird & Dinkova‐Kostova, 2011) and activates anti‐inflammatory and anti‐oxidant enzymes, including heme‐oxygenase‐1 (HO‐1) and NAD(P)H‐quinone oxidoreductase 1 (NQO1) (Feng et al., 2019). Nrf2 activation can mitigate neurodegenerative damage in Parkinson's disease, multiple sclerosis, stroke, and SCI (Benarroch, 2017; Samarghandian et al., 2020), while impaired Nrf2 signaling may lead to oxidative stress as in Friedreich's ataxia (Paupe et al., 2009).

We hypothesized that EA would exert neuroprotective effects on the CNS after SCI, acting through ApoE and Nrf2 to mitigate inflammation and oxidative stress. We compared functional recovery after SCI in wild‐type vs. ApoE‐deficient mice, and examined the potential effects of EA alone and in combination with exogenous ApoE mimetic peptide.

## 2 MATERIALS AND METHODS

### 2.1 Animals

Adult female C57BL/6 wild‐type (WT) mice and homologous *ApoE* ^–/–^ mice (2–3 months old, 18–20 g) were used in experiments. Animals were obtained from the Experimental Animal Center of Chongqing Medical University. Mice were housed in standard cages located in a specific pathogen‐free room with a 12‐h light/dark cycle. The room temperature was maintained at 22–24℃ and relative humidity at 65–75%, and animals had free access to food and water. All experiments were carried out in strict accordance with “Guiding Opinions on the Treatment of Experimental Animals” (2006) from the Ministry of Science and Technology of the People's Republic of China. All procedures were approved by the Ethics Committee of Chongqing Medical University.

WT and *ApoE* ^–/–^ mice were randomly divided into groups (*n* = 18 mice/group): Sham group, SCI group, SCI+EA group (abbreviated to EA group), SCI+COG112 group (abbreviated to COG112 group), and SCI+EA+COG112 group (abbreviated to EA+COG112 group). Mice in the sham group received only laminectomy. Mice in the SCI group underwent L1 SCI. Mice in the EA group performed *zusanli* and *sanyinjiao* EA therapy the day after SCI, 10 min daily for 6 consecutive days with one day off, until the mice were killed. Mice in the COG112 group were intraperitoneally injected with *ApoE*‐Mimetic Peptides (COG112, dissolved in lactated Ringer's buffer, 1 mg/kg) 3 h after surgery, twice daily, until the mice were killed. Mice in the EA+COG112 group received both EA and COG112 (Table 1). Mice in sham, SCI, and EA groups received the same volume of lactated Ringer's buffer as vehicle control. Animals were sacrificed at the end of week 4 after SCI for histological and biochemical examination.

**TABLE 1:** Animals used for experiments

Groups Number Species	Sham	SCI	EA	COG112	EA+COG112
WT mice	18	18	18	18	18
*ApoE* −/− mice	18	18	18	18	18

### 2.2 Surgical procedure

Mice were anesthetized by injecting 1% sodium pentobarbital (80 mg/kg) intraperitoneally. SCI was performed as described (Paterniti et al., 2018). The skin was incised with a scalpel along the midline of the back to expose the paravertebral muscles. The superficial and deep paravertebral muscles parallel to the spine processes were removed. T12‐L2 spine process and the bilateral laminae were dissected out by surgical forceps without disruption to the dura. Aneurysm clip was inserted around the exposed spinal cord for 25 s to generate an extradural compression of 20× g pressure that causes an acute compression injury. After surgery, muscles and skin were sutured in layers. Mice were kept warm on heating pads until they fully recovered from the anesthesia, and were injected intramuscularly with penicillin (2,000,000 U/kg/day per mouse) for three days after surgery to prevent infection.

### 2.3 EA

Acupuncture at *zusanli* and *sanyinjiao* can improve axonal growth and spinal cord plasticity and inhibit inflammatory reaction after SCI (Hong et al., 2021; Huang et al., 2015). Starting on the day after surgery, the mice in EA group and EA+COG112 group were treated with EA stimulation 10 min daily for 6 consecutive days, followed by 1 day off, and lasted for 4 weeks. EA treatment was performed at the bilateral acupoints *zusanli* (ST 36, located at the anterior aspect of the hindlimb, 2 mm directly below the knee joint) and *sanyinjiao* (SP 6, located posterior to the tibia, 3 mm above the medial malleolus). Stainless steel needles (0.25 mm × 13 mm, Jiangsu Medical Instruments Inc., China) were inserted into the acupoints with a depth of 3–5 mm below the skin. Then, the needle handles were linked to the output terminals of an electronic acupuncture instrument (SDZ‐II, Suzhou Medical Products, Suzhou, China), which offered a pattern of parse‐dense waves (60 Hz for 1.05 s and 2 Hz for 2.85 s, alternately). The current intensity was ≤ 5 μA in the animals’ body, which was strong enough to induce a mild twitch in the hind limbs.

### 2.4 Exogenous ApoE treatment

The ApoE‐Mimetic Peptides, COG112 (acetyl‐RQIKIWFQNRRMKWKKCLRVRLASHLRKLRKRLL‐amide) as synthesized by Gill Biochemicals (Shanghai, China) and purified by high‐performance liquid chromatography to a purity of >95%. COG112 was dissolved in lactated ringer's buffer and injected intraperitoneally (1 mg/kg) 3 h after surgery every other day for 4 weeks (F. Q. Li et al., 2010).

### 2.5 Hind limb locomotor function

The Basso mouse scale (BMS) is a method to score hind limb locomotor function in spinal cord injured mice (Basso et al., 2006). The score ranges from 0 to 9, in which 0 denotes complete paralysis and 9 denotes normal movements. Two investigators blinded to experimental groups scored the hind limb locomotor function of mice according to ankle joint mobility, coordination, paw status, trunk stability, and tail posture. Each mouse was observed for 5 min in an open field, and the average of both scores was taken as the final score. This test was performed on days 1, 3, 7, 14, and 28 after SCI.

### 2.6 Tissue preparation

At day 28 after injury, the mice were deeply anesthetized and transcardially perfused with 0.01 M phosphate‐buffered saline (PBS) followed by 4% paraformaldehyde solution in 0.01 M PBS at room temperature. A 1 cm‐long spinal cord segment [centered at the injury epicenter (L1), the spinal cord was 0.5 cm above and below the injury epicenter] was harvested and fixed for 24 h with the 4% paraformaldehyde solution in 0.1 M PBS at 4℃. Then the tissue was completely dehydrated through a graded ethanol series, permeabilized with xylene, embedded in paraffin and cut into 6 μm‐thick sections for histological analysis.

### 2.7 Histology

Tissue sections were deparaffinized with xylene and immersed in hematoxylin solution for 3 min, followed by a quick rinse in distilled water. Then the slides were differentiated in HCl/95% alcohol (1:50) solution for 10 s. After washing in distilled water for 5 min, the sections were stained with 0.5% eosin for 10 s, then dehydrated through a gradient ethanol series from 95% to 100% for 3 min, and cleared by xylene for 1 min. The sections were examined under a light microscope (BX 53, Olympus, Tokyo, Japan).

Sections were scored blindly by two investigators based on edema, hemorrhage, tissue cavity, neuronal apoptosis or necrosis, and inflammatory cell infiltration. A score of 0 was given for no or minor pathology; 1, limited pathology; 2, intermediate pathology; 3, prominent pathology; or 4, extensive pathology.

### 2.8 Immunofluorescence

For immunofluorescence staining, tissue sections were deparaffinized by xylene, rinsed, and blocked with goat serum (Boster Biological Technology, Pleasanton, California, USA) at 37°C in a humidified atmosphere for 1 h. Then sections were incubated overnight at 4°C with rabbit monoclonal anti‐ApoE (1:400; Abcam, Cambridge, UK) or rabbit polyclonal anti‐Nrf2 (1:400; Abcam). The sections were rinsed with 0.01 M PBS and incubated with goat IgG conjugated to Alexa 492 (1:200; Earthox, Shanghai, China) for 1 h at 37°C in a dark humidified chamber. Finally, nuclei were stained with 4′,6‐diamidino‐2‐phenylindole (Beyotime Bio, Shanghai, China) for 5 min. After a final rinse, coverslips were mounted with an antifade mounting medium (Beyotime Bio), and sections were observed under a fluorescence microscope.

### 2.9 Transmission electron microscopy

Mice were deeply anesthetized and transcardially perfused with 0.01 M PBS, followed by a mixture of 4% paraformaldehyde and 2.5% glutaraldehyde (1:1). A 0.5 cm‐long spinal cord segment [centered at the injury site (L1), the spinal cord was 0.25 cm above and below the injury epicenter] was harvested and fixed with 2.5% glutaraldehyde at 4℃. Samples were placed in 1% osmium tetroxide and dehydrated through ascending graded solutions of ethanol and acetone. Following embedding in Epon‐Araldite resin, tissues were cut into ultrathin sections, placed on copper grids, stained with uranyl acetate and lead citrate, and imaged on a Philips EM420 electron microscope (Philips Electron Optics, Amsterdam, The Netherlands). Image Pro Plus 6.0 Software (Media Cybernetics, Silver Spring, MD, USA) was used to quantify the ratio of demyelinated axons to total axons.

### 2.10 Western blot analysis

Protein levels of ApoE, Nrf2, HO‐1, and NQO1 were determined at 28 days post‐injury. Mice were deeply anesthetized and transcardially perfused with 0.01 M PBS. A 1 cm‐long spinal cord segment [centered at the injury site (L1), the spinal cord was 0.5 cm above and below the injury epicenter] was harvested and grounded up in a mixture of RIPA lysis buffer (Beyotime Bio) containing 1% protease inhibitor on ice. The homogenate was centrifuged at 12,000 g for 15 min at 4°C. The supernatant was diluted with 5× protein loading buffer (Beyotime Bio), and heated at 95°C for 5 min. Equal amounts of protein (40 μg) were loaded for each sample and separated on a 10% SDS polyacrylamide gel electrophoresis system, then transferred to polyvinylidene fluoride membranes (General Electric Company, New York, USA). After blocking non‐specific binding sites with 5% skim milk for 2 h at room temperature, the membranes were incubated overnight with the following antibodies at 4°C: rabbit monoclonal anti‐ApoE (1:1000, Abcam), rabbit polyclonal anti‐Nrf2 (1:1000, Abcam), rabbit monoclonal anti‐NQO1(1:10000, Abcam), rabbit polyclonal anti‐HO‐1 (1:2000, Abcam); and rabbit anti‐β‐actin (1:5000, Cell Signaling Technology, Danvers, MA, USA). Horseradish peroxidase‐labeled goat anti‐rabbit IgG (1:100, Boster) was then incubated for 1 h at 37°C. Protein bands were exposed for 1 min with Enhanced Chemiluminescence detection reagent (4A Biotech, Beijing, China). The intensity of all protein bands was measured using Image J 2x (NIH, Bethesda, MD, USA) and normalized to the level of β‐actin. All western blot experiments were performed in triplicate.

### 2.11 Quantitative reverse transcription‐polymerase chain reaction (qRT‐PCR)

Levels of mRNAs encoding the proinflammatory cytokines TNF‐α, IL‐6, or IL‐1β or the anti‐inflammatory cytokines IL‐10 and transforming growth factor‐β1 (TGF‐β1) were determined at 28 d post‐injury. Mice were transcardially perfused with 0.01 M PBS as above. Standardized areas of spinal cord tissue (5 mm cephalad and 5 mm caudal to the lesion center) were harvested. Total mRNA was extracted using RNAiso Plus (TaKaRa, Beijing, China) according to the manufacturer's instructions. Reverse transcription to cDNA was performed following the reaction protocol provided with the PrimeScript^®^ RT reagent Kit with gDNA Eraser (TaKaRa). The reaction mixture contained Fast SYBR Green master mix (TaKaRa), 10 mM forward and reverse primers (TaKaRa), RNase‐free water, and cDNA in a total reaction volume of 10 μl. The following primer sequences were used: *TNF‐α* forward, 5′‐GCCAACGGCATGGATCTCAA‐3′; *TNF‐α* reverse, 5′‐TGACGGCAGAGAGGAGGTTGA‐3′; *IL‐6* forward, 5′‐CTTGGGACTGATGCTGGTGAC‐3′; *IL‐6* reverse, 5′‐TTCTCATTTCCACGATTTCCCA‐3′; *IL‐1β* forward, 5′‐GCTGCTTCCAAACCTTTGACC‐3′; *IL‐1β* reverse, 5′‐AATGAGTGATACTGCCTGCCTGA‐3′; *IL‐10* forward, 5′‐TTGCCAAGCCTTATCGGAAAT‐3′; *IL‐10* reverse, 5′‐TGAGGGTCTTCAGCTTCTCACC‐3′; *TGF‐β1* forward, 5′‐GAGGCGGTGCTCGCTTTGTA‐3′; *TGF‐β1* reverse, 5′‐CGTTGTTGCGGTCCACCATTA‐3′; *β‐actin* forward, 5′‐AGATTACTGCTCTGGCTCCTAGC‐3′; and *β‐actin* reverse, 5′‐ACTCATCGTACTCCTGCTTGCT‐3′. Quantitative PCR was conducted using the CFX 96 real‐time PCR detection system (Bio‐Rad, Hercules, CA, USA) under the following cycling conditions: 95℃ for 30 s, followed by 40 cycles at 95°C for 5 s, and 60°C for 30 s. Levels of mRNA were determined using the 2^−ΔΔCT^ method and normalized to levels of *β‐actin* mRNA.

### 2.12 Statistical analysis

All statistical analyses were performed by GraphPad Prism 5.01 (GraphPad Software, San Diego, CA, USA). Data are expressed as mean ± SD. Inter‐group differences on Basso scores were assessed for significance using ANOVA of repeated measurements, while other inter‐group differences were assessed using one‐way ANOVA and Bonferroni's post hoc multiple comparison test. Differences were considered statistically significant when *p* < .05.

## 3 RESULTS

### 3.1 EA combined with exogenous ApoE promotes recovery of hindlimb function and neuronal morphology after SCI in mice

**FIGURE 1:**
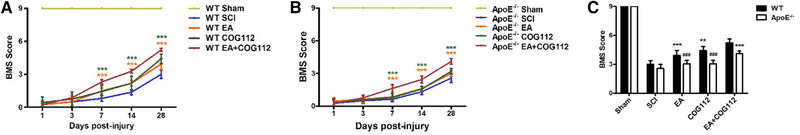
Assessment of locomotor function in C57BL/6 WT and *ApoE* ^–/–^ mice subjected to spinal cord injury was assessed by Basso mouse scale (BMS; *n* = 18 mice per group). (a) Locomotor function of the hind limb was assessed in WT mice over time. (b) Locomotor function of the hind limb was assessed in *ApoE* ^–/–^ mice over time. (c) Total scores were assessed in WT and *ApoE* ^–/–^ mice at 28 days post‐injury. All data are mean ± SD. Abbreviations: ApoE, apolipoprotein E; EA, electroacupuncture; SCI, spinal cord injury; WT, wild type. ^**^ *p* < .01, ^***^ *p* < .001 vs. WT EA+COG112 group. ^###^ *p* < .001 vs. *ApoE* ^–/–^ EA+COG112 group

As shown in Figure 1, BMS scores of WT and *ApoE* ^–/–^ mice in sham groups displayed normal physical movements. No statistically significant differences were found in the baseline studies among SCI, EA, COG112, and EA+COG112 groups at one day after surgery (Figure 1a,b). The mice became spastic, paralyzed, and incontinent. The mean BMS in all groups were increased with the duration of time. The locomotion score in WT EA+COG112 group mice was significantly higher compared with that of the other WT mice at 28 days after injury. The mice could stand, but not harmoniously. The locomotion score in *ApoE* ^–/–^ EA+COG112 group mice was significantly higher compared with that of the other *ApoE* ^–/–^ mice at 28 d after injury. The mice could stand occasionally. The locomotion score in *ApoE* ^–/–^ EA group was significantly lower than that of the *ApoE* ^–/–^ COG112 and EA+COG112 groups at 28 days after injury (Figure 1c). In other words, the presence of ApoE markedly strengthened the ability of EA to restore locomotor function, whereas lack of ApoE significantly weakened its ability to do so.

**FIGURE 2:**
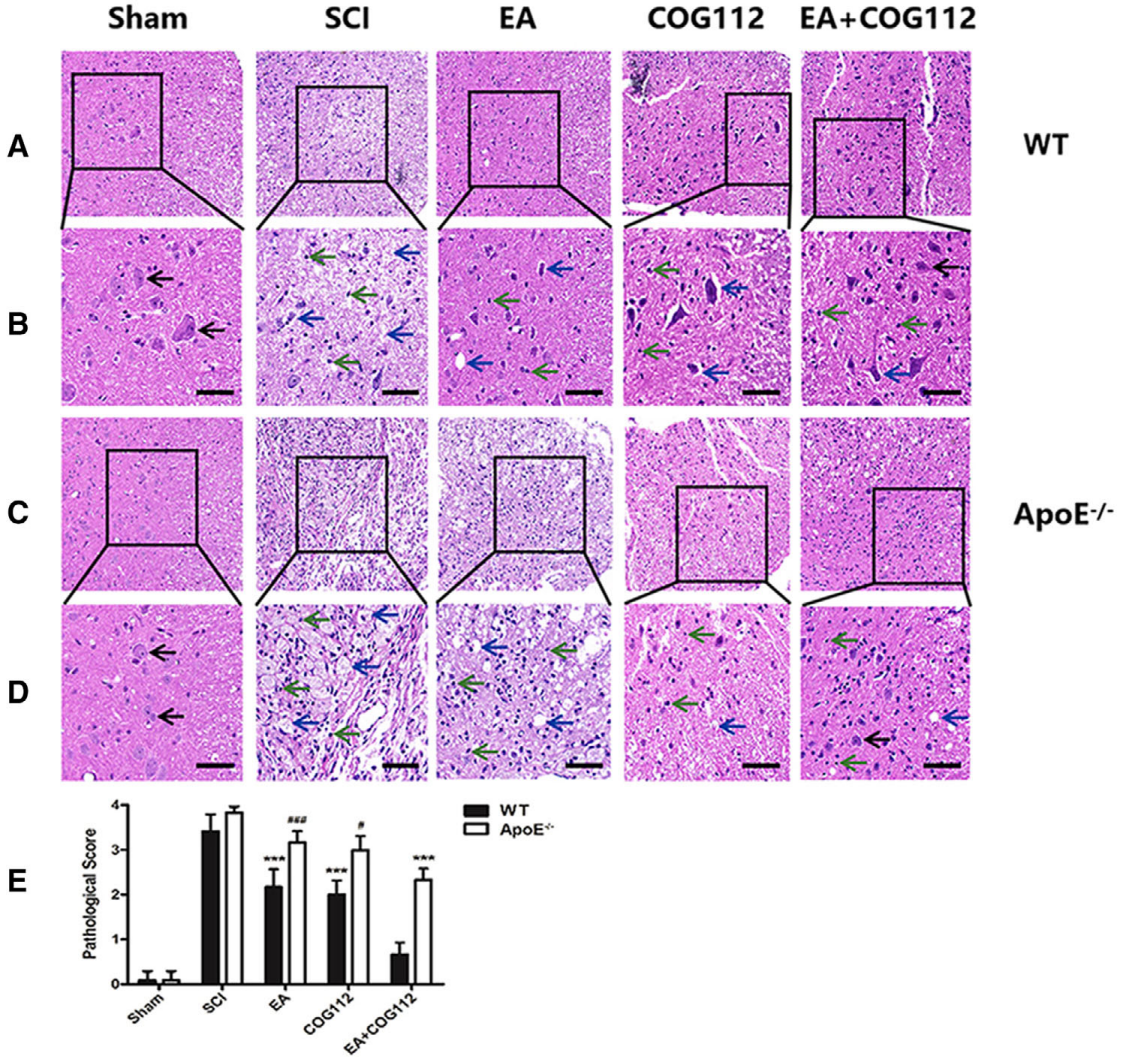
Micrographs of hematoxylin‐ and eosin‐stained spinal cord tissue near the epicenter of damage at 28 days after SCI (*n* = 6 mice per group). (a) WT mice. (b) The local enlarged pictures from panel (a). (c) *ApoE* ^−/−^ mice. (d) The local enlarged pictures from panel (c). (e) Quantitative analysis of pathology scores from experiments. Scale bar: (a, c) 50 μm; (b, d) 20 μm. Black arrows point to normal neurons; blue arrows, to apoptotic or necrotic neurons; and green arrows, to inflammatory cytokines. All data are mean ± SD. Abbreviations: ApoE, apolipoprotein E; EA, electroacupuncture; SCI, spinal cord injury; WT, wild type. ^***^ *p* < .001 vs. WT EA+COG112 group. ^#^ *p* < .05, ^###^ *p* < .001 vs. *ApoE* ^–/–^ EA+COG112 group

Histochemistry revealed significant damage to the spinal cord at 28 days after injury in SCI animals, including structural disorganization, tissue cavities, neuronal apoptosis or necrosis, and inflammatory infiltration. Similar signs of damage, but less severe, were seen in EA, COG112, and EA+COG112 groups (Figure 2a–d), with EA+COG112 animals in WT mice ultimately showing significantly lowest pathological scores (Figure 2e).

**FIGURE 3:**
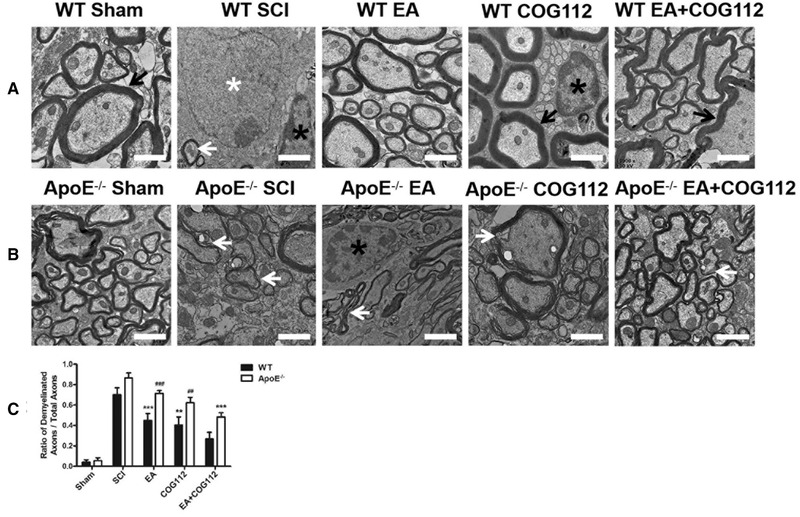
Transmission electron micrographs showing the ultrastructure of the spinal cord at 28 d post‐injury (*n* = 6 mice per group). (a) WT mice. (b) *ApoE* ^–/–^ mice. (c) Quantification of the ratio of demyelinated axons to total axons in the experiments. Scale bar = 1 μm. White arrows: demyelinated axons; black arrows: normal axons. White asterisk: neuron nucleus; black asterisks: oligodendrocyte nuclei. All data are mean ± SD. Abbreviations: ApoE, apolipoprotein E; EA, electroacupuncture; SCI, spinal cord injury; WT, wild type. ^**^ *p* < .01, ^***^ *p* < .001 vs. WT EA+COG112 group. ^##^ *p* < .01, ^###^ *p* < .001 vs. *ApoE* ^–/–^ EA+COG112 group

Myelination allows rapid nerve impulse conduction (Franssen, 2019), so we examined the ultrastructure of axons and myelin sheaths in the spinal cord, as well as the neurons and glial cells. In the sham WT mice, axons were tightly wrapped in layers of compact myelin sheaths. The myelin sheaths were degraded, loosened and separated from axons, and the neuron was swollen in the SCI group. The demyelination in *ApoE* ^–/–^ mice was worse than that in the WT mice. After treatment, axons were wrapped by oligodendrocytes and the demyelination was reversed in EA, COG112, and EA+COG112 groups (Figure 3a,b), with EA+COG112 reversing this demyelination to a significant extent in WT mice (Figure 3c).

Thus, EA combined with exogenous ApoE significantly enhanced hindlimb locomotor function, reduced neural tissue loss and inflammatory response, and suppressed myelin degeneration and axonal demyelination in WT and *ApoE* ^–/–^ mice model of SCI.

### 3.2 The expressions of ApoE and Nrf2 in WT and ApoE^–/–^ mice model of SCI

**FIGURE 4:**
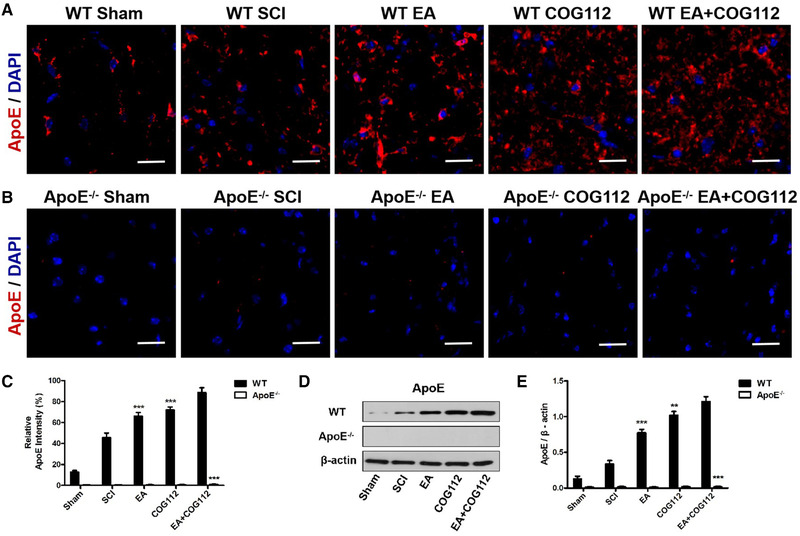
Expression levels of ApoE at 28 d after SCI in spinal cord tissue from WT and *ApoE* ^–/–^ mice (*n* = 6 samples per group). (a) Immunofluorescence staining of ApoE in WT mice. (b) Immunofluorescence staining of ApoE in *ApoE* ^–/–^ mice. Scale bar = 20 μm. Cell nuclei were stained with DAPI (blue). (c) Quantitative analysis of relative ApoE fluorescence intensity in experiments. (d) Western blot showing the expression of ApoE in the spinal cord from WT and *ApoE* ^–/–^ mice. β‐actin served as the internal control. (e) Quantitative analysis of relative ApoE protein expression in experiments in panel (d). All data are mean ± SD. Abbreviations: ApoE, apolipoprotein E; DAPI: 4′,6‐diamidino‐2‐phenylindole; EA, electroacupuncture; SCI, spinal cord injury; WT, wild type. ^**^ *p* < .01; ^***^ *p* < .001 vs. WT EA+COG112 group

**FIGURE 5:**
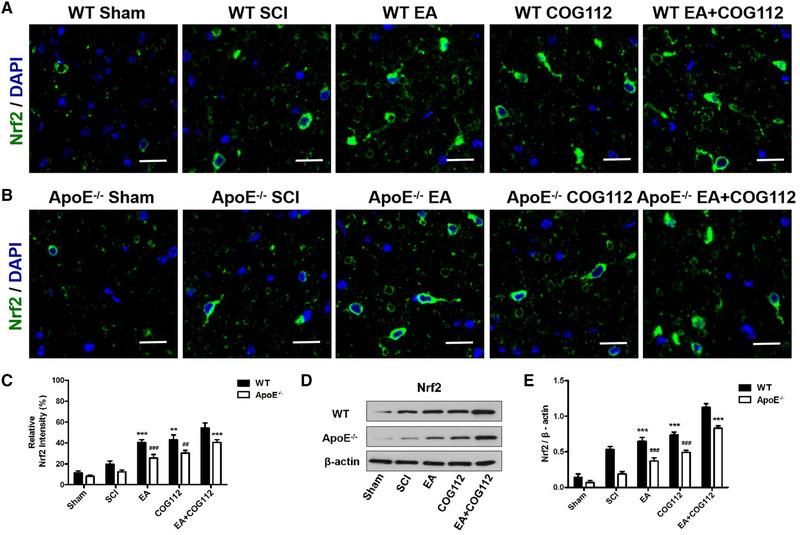
Expression levels of Nrf2 at 28 days after SCI in spinal cord tissue from WT and *ApoE* ^–/–^ mice (*n* = 6 samples per group). (a) Immunofluorescence staining of Nrf2 in WT mice. (b) Immunofluorescence staining of Nrf2 in *ApoE* ^–/–^ mice. Scale bar = 20 μm. Cell nuclei were stained with DAPI (blue). (c) Quantitative analysis of relative Nrf2 fluorescence intensity in experiments. (d) Western blot showing the expression of Nrf2 in the spinal cord from WT and *ApoE* ^–/–^ mice. β‐actin served as the internal control. (e) Quantitative analysis of relative Nrf2 protein expression in experiments in panel (d). All data are mean ± SD. Abbreviations: ApoE, apolipoprotein E; DAPI, 4′,6‐diamidino‐2‐phenylindole; EA, electroacupuncture; Nrf2, nuclear factor erythroid 2‐related factor; SCI, spinal cord injury; WT, wild type. ^***^ *p* < .001 vs. WT EA+COG112 group. ^###^ *p* < .001 vs. *ApoE* ^–/–^ EA+COG112 group

Histochemistry also revealed the effect of EA on ApoE and Nrf2 in WT and ApoE^–/–^ mice model of SCI. EA induced the activation of ApoE and Nrf2, and the WT EA+COG112 group expressed the highest levels of ApoE and Nrf2 (Figures 4 and 5). The *ApoE* ^–/–^ mice showed the opposite. These results directly implicate ApoE and Nrf2 as a downstream mediator of EA after SCI.

### 3.3 EA‐mediated improvement in inflammatory response after SCI depends on ApoE

**FIGURE 6:**
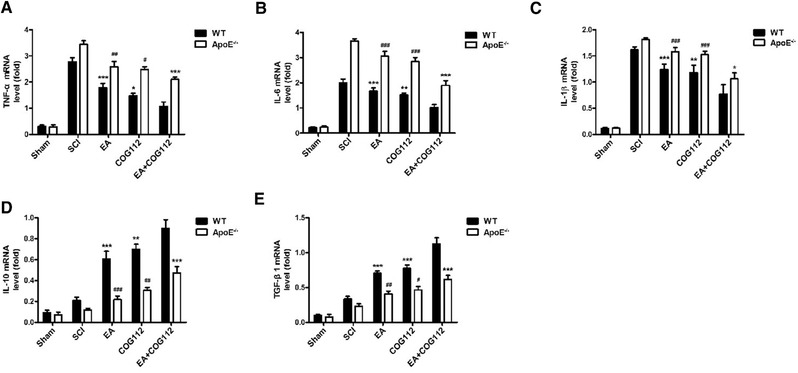
Levels of mRNA encoding anti‐ or pro‐inflammatory cytokines in spinal cord tissue from WT and *ApoE* ^–/–^ mice at 28 d after spinal cord injury (*n* = 6 samples per group). (a–c) Quantitative analysis of pro‐inflammatory cytokine mRNA levels. (d,e) Quantitative analysis of anti‐inflammatory cytokine mRNA levels. All data are mean ± SD. Abbreviations: ApoE, apolipoprotein E; EA, electroacupuncture; IL‐1β, interleukin‐1β; IL‐6, interleukin‐6; IL‐10, interleukin‐10; SCI, spinal cord injury; TGF‐β1, transforming growth factor‐β1; TNF‐α, tumor necrosis factor; WT, wild type. ^*^ *p* < .05; ^**^ *p*< .01; ^***^ *p* < .001 vs. WT EA+COG112 group. ^#^ *p* < .05, ^##^ *p* < .01, ^###^ *p* < .001 vs. *ApoE* ^–/–^ EA+COG112 group

Next, we asked whether the ApoE‐dependent recovery induced by EA involves down‐regulation of the pro‐inflammatory cytokines TNF‐α, IL‐1β, or IL‐6, and up‐regulation of the anti‐inflammatory cytokines IL‐10 and TGF‐β1. Indeed, the WT EA+COG112 group expressed the lowest mRNA levels of pro‐inflammatory cytokines, but the highest levels of anti‐inflammatory cytokines (Figure 6). The *ApoE* ^–/–^ mice showed the opposite.

Exogenous ApoE significantly down‐regulated pro‐inflammatory cytokine mRNAs and up‐regulated anti‐inflammatory cytokine mRNAs in WT and *ApoE* ^–/–^ animals.

### 3.4 EA‐mediated improvement in oxidative stress after SCI depends on ApoE

**FIGURE 7:**
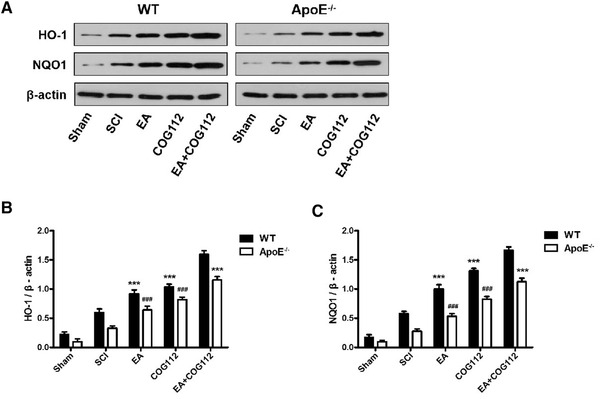
Levels of proteins in the Nrf2/HO‐1 signaling pathway in spinal cord tissue from WT and *ApoE* ^–/–^ mice at 28 days after spinal cord injury (*n* = 6 samples per group). All data are mean ± SD. Abbreviations: ApoE, Apolipoprotein E; EA, electroacupuncture; HO‐1, heme‐oxygenase‐1; NQO1, NAD(P)H‐quinone oxidoreductase 1; SCI, spinal cord injury; WT, wild type. ^***^ *p* < .001 vs. WT EA+COG112 group. ^###^ *p* < .001 vs. *ApoE* ^–/–^ EA+COG112 group

We examined whether the ApoE‐dependent recovery induced by EA involves the Nrf2/ NQO1/HO‐1 signaling pathway, which mitigates oxidative stress in certain neurodegenerative diseases. Expressions of NQO1 and HO‐1 proteins positively correlated with ApoE expression (Figure 7): the expressions were highest in WT EA+COG112 animals and lowest in *ApoE* ^–/–^ animals. Thus, the expressions of the two proteins also correlated with the extent of functional and neuromorphological recovery after SCI. These results suggest that EA induces Nrf2/NQO1/HO‐1 signaling via an ApoE‐dependent pathway, thereby mitigating oxidative damage after SCI.

## 4 DISCUSSION

Our previous work found that EA can reduce inflammation and oxidative stress reactions via up‐regulating expression of ApoE, phosphorylated extracellular regulatory protein kinase, and Nrf2/HO‐1 and inhibiting IL‐1β and NF‐κB expression at the early stage after SCI in C57BL/6 wild‐type mice (Dai et al., 2019). Our present studies in the knockout (*ApoE* ^–/–^) mouse model of SCI further suggest that EA can improve locomotor dysfunction, reduce inflammatory response and oxidative stress, and promote remyelination. It can also inhibit inflammatory responses and oxidative stress through activation of the Nrf2/HO‐1 pathway. These effects appear to depend on ApoE.

SCI consists of two pathological processes that include immediate primary mechanical injury and subsequent secondary injury, which exacerbates neurological deficits and outcomes (Yip & Malaspina, 2012). Inflammatory responses and oxidative stress are two major types of secondary injury (Heo et al., 2020), during which inflammatory cells such as macrophages, microglia and neutrophils (Kumar et al., 2018) trigger the release of pro‐inflammatory cytokines TNF‐α, IL‐1β and IL‐6 (Nakamura et al., 2003), leading to cellular necrosis or apoptosis (McPhail et al., 2004)^.^ In addition, neurons of the dorsal horn in the spinal cord release the TGF‐β 1, an anti‐inflammatory transforming growth factor (Xiyang et al., 2014). The increased levels of IL‐6 and IL‐1 were recognized as the main cause of the severity of neurological disorders in the Nrf2 knockout mice after SCI (Mao et al., 2012). Inflammatory cells also release excess reactive oxygen and nitrogen species (C. Wang et al., 2019), which cause DNA oxidative damage, protein oxidation and lipid peroxidation (Ahuja et al., 2017), exacerbating necrosis and apoptosis of neurons and glial cells (Alizadeh et al., 2019).

EA, widely used in China, has shown good therapeutic efficacy against SCI and its sequelae (Meng et al., 2015; Zhou et al., 2006). EA mitigates SCI by reducing edema, inflammatory response, lipid peroxidation and excitatory amino acid toxicity, thus promoting neuronal survival as well as axonal regeneration and remyelination (Ding et al., 2013; Jin et al., 2019; Zhao et al., 2017). Neuroanatomical and neurological evidence demonstrated that the involvement of the nervous system is critical for the acupuncture effects. The abundant meridians and acupoints distribute in human nerve endings (A. H. Li et al., 2004). *Zusanli* (located at the anterior aspect of the hindlimb, 2 mm directly below the knee joint) and *sanyinjiao* (located posterior to the tibia, 3 mm above the medial malleolus) can accelerate the conduction of nerve impulses (Huang et al., 2015).

Except for ST36 and SP 6, there are many other acupoints which benefit spinal cord injury. Ding et al. (2013) found Governor Vessel acupoints can also promote neuronal survival and axonal regeneration of injured spinal cord (Xu et al., 2021). Wong et al. (2003) demonstrated that EA at SI3 and BL62 in conjunction with auricular acupoints produced enhanced recovery of bladder function in patients with SCI. Jia‐Ji acupoints may also improve locomotor function by promoting autophagy flux and inhibiting necroptosis (Hongna et al., 2020). In a word, EA plays a key role in the recovery of SCI. The present study confirms these findings and extends them by showing that EA acts through ApoE and the Nrf2/HO‐1 pathway. ApoE is an important therapeutic target of EA against SCI and the effect of EA depends on the ApoE.

ApoE has been shown to exert anti‐inflammatory, anti‐oxidative and anti‐apoptotic properties (Laskowitz et al., 1997, 1998), making it an important modulator of neuronal repair and remodeling in trauma and diseases of the central nervous system (Teng et al., 2017). Loss of ApoE aggravates the inflammatory response and oxidative stress as well as increases neural apoptosis, thus retarding the recovery of locomotor and neurological functions after SCI (Yang et al., 2018). In addition, ApoE has an important role in the remyelination process after experimental demyelination in animals (Boyles et al., 1985). Abnormalities in endogenous ApoE could interfere with the metabolism of myelin lipids. We showed that the neuroprotective effects of EA were weakened in the absence of ApoE (as a result of gene deletion) and strengthened in its presence (through supplementation with ApoE‐like peptide COG112). Our findings are consistent with the reported ability of COG112 to improve neurological and histological outcomes following rat with SCI (R. Wang et al., 2014), which is associated with inhibition of NF‐κB‐induced inflammation and demyelination in the spinal cord (F. Q. Li et al., 2006; Singh et al., 2011). These data strongly suggest that EA acts via ApoE, which may justify therapies targeting this protein.

Our results identify at least one way in which EA reduces inflammation via ApoE: it down‐regulates the pro‐inflammatory cytokines TNF‐α, IL‐6, and IL‐1β, while up‐regulating the anti‐inflammatory cytokines IL‐10 and TGF‐β1. The pro‐inflammatory cytokines are released immediately by microglia and other neurons, as well as endothelial cells after SCI. After 3–4 days of injury, peripheral monocytes and macrophages are recruited to the injury site and reaches peak levels after 7–10 days of injury and persists for several months (Samarghandian et al., 2015). Persistence inflammatory responses increase the risk of secondary injury progression after SCI (Schwab & Caroni, 1988). Indeed, ApoE‐deficiency on its own, significantly up‐regulates pro‐inflammatory cytokines after SCI (Pandey et al., 2007), and our addition of exogenous COG112 enhanced the effects of EA.

Not only that, our results also identify at least one way in which EA reduces oxidative stress via ApoE: it induces ApoE to activate anti‐oxidative signaling by Nrf2, which has been shown to play a crucial anti‐oxidative role after SCI in animal models (Feng et al., 2019; Molagoda et al., 2020). Several studies have reported the role of Nrf2 signaling pathways in the mice models of SCI. The Nrf2/HO‐1/NQO1 signaling axis serves as a robust anti‐oxidant pathway in stress‐activated and age‐related diseases (Zhang et al., 2019). Z. Li et al. (2019) found that inhibition of triggering receptor expressed on myeloid cells 1 significantly decreased inflammation and oxidative stress by Nrf2/HO‐1 expression. The Nrf2/HO‐1 signaling pathway was associated with the anti‐inflammatory effect of IL‐10 (Syapin, 2008). Their association may help explain why ApoE and Nrf2 levels correlate with each other (Mezera et al., 2015) and why they show similar anti‐oxidant properties (Pandey et al., 2007), as we observed in the present study. Lack of ApoE reduces the expression of Nrf2 and HO‐1 after SCI, resulting in increased oxidative stress (Yang et al., 2018).

Although our findings hold great potential for treating spinal cord injury patients, there are some limitations to the present study. Currently, only *zusanli* and *sanyinjiao* are not enough to treat SCI patients and there are no standard acupoint prescription in clinic. Future studies should explore other more effective acupoints for SCI. In addition, more proteins and pathways, which can regulate the association between ApoE and Nrf2 should be examined in CNS (Pomeshchik et al., 2014).

## 5 CONCLUSIONS

EA may be an effective treatment to improve motor dysfunction, inflammation, and demyelination after SCI, and it appears to exert these effects by up‐regulating anti‐inflammatory cytokines, down‐regulating pro‐inflammatory cytokines, and mitigating oxidative stress, all through an ApoE‐dependent mechanism.

## CONFLICT OF INTEREST

All authors claim that there are no conflicts of interest/competing interests.

## AUTHOR CONTRIBUTIONS

Ni Dai and Chenglin Tang performed the whole experiments and molecular studies and drafted the manuscript. Hui Liu performed the molecular biology study. Siqin Huang funded and conceived the study, participated in the study design, and revised the manuscript. All authors read and approved the final version of the manuscript.

### PEER REVIEW

The peer review history for this article is available at https://publons.com/publon/10.1002/brb3.2328

## Data Availability

The datasets used and/or analyzed during the current study are available from the corresponding author on reasonable request.
